# Time series forecasting methods in emergency contexts

**DOI:** 10.1038/s41598-023-42917-1

**Published:** 2023-09-26

**Authors:** P. Villoria Hernandez, I. Mariñas-Collado, A. Garcia Sipols, C. Simon de Blas, M. C. Rodriguez Sánchez

**Affiliations:** 1 Department of Electronics, Rey Juan Carlos University, Madrid, Spain; 2 Department of Statistics and Operations Research and Mathematics Didactics, University of Oviedo, Oviedo, Spain; 3 Department of Applied Mathematics, Materials Science and Engineering and Electronic Technology, Rey Juan Carlos University, Madrid, Spain; 4 Department of Computer Sciences and Statistics, Rey Juan Carlos University, Madrid, Spain

**Keywords:** Statistics, Electrical and electronic engineering

## Abstract

The key issues in any fire emergency are recognising fire hotspots, locating the emergency intervention team (EI), following the evolution of the fire, and selecting the evacuation path. This leads to the study and development of HelpResponder, a solution capable of detecting the focus of interest in hostile spaces derived from fire due to high temperatures without visibility. A study is conducted to determine which model best predicts measured $\text {CO}_2$ levels. The variables used are temperature, humidity, and air quality, obtained from sensors installed in a fire tower. The statistical methods applied, namely ARIMAX, KNN, SVM, and TBATS, allow the adjustment and modelling of the variables. Explanatory variables with temporal structure are incorporated into SVM, a new improvement proposal. Moreover, combining different models showed the best efficiency in forecasting. In fact, another contribution of our work lies in offering a small-scale prediction system that is specifically designed to save batteries. The system has been tested and validated in a hostile environment (building), simulating real emergency situations. The system has been tested and validated in several hostile environments, simulating real emergency situations. It can help firefighters respond faster in an emergency. This reduces the risks associated with the lack of information and improves the time for tactical operations, which could save lives.

## Introduction

Cities are one of the vortex in the Sustainable Development Goals of the United Nations’ 2030 Agenda which aims to achieve sustainable and resilient cities^1^. This implies a more efficient use of resources improving sustainability development. Furthermore, the population growth in cities, which is observed in the fact that $70\%$ of the population now lives in urban areas^2^ and is expected to increase up to $85\%$ by 2050^3^, implies a constant need in this line. One of the key points in smart cities, is to provide resilient solutions related to emergencies that permits return to the ordinary state as soon as possible^4^. Digitalization is a useful tool in urban transformation of emergency services capable to provide information in real time, improving the process efficiency. For such a purpose, monitoring, evaluating, integration and management of assets conform a elaborated strategy.

Related to emergency services, the study of fire victims in Spain conducted by the Mapfre Foundation in collaboration with the Professional Association of Fire Technicians (APTB)^5^, The number of fatalities in fires and explosions in Spain in 2019 was 165, of which 158 corresponded to deaths in fires. These data represent an increase of $34\%$ over the previous year, and $97\%$ of total fire deaths are caused by direct exposure to fire (smoke inhalation, burns, overexertion or stress, or becoming trapped). The big problem is, above all, the inhalation of toxic gases. The characteristics that give rise to these situations occur mainly in enclosed spaces such as galleries and tunnels. Despite the concerns of the Prevention and Safety teams do not find a definitive solution to locate the source of the emergency^6^. The fire detection systems used in some works find the problem that they must be close to the source of the fire and are not always reliable because smoke and the characteristics of some elements (colors or textures)—such as light bulbs or fluorescent lamps—do not necessarily indicate a fire^7,8^.

On the one hand, to solve this problem, it would be necessary to monitor the useful data during these situations (type of intervention, type of environment, environmental parameters, parameters associated with victims or intervention personnel). On the other hand, it is important to guide the emergency team inside the site in severely hostile conditions by following the action protocol. Most of the solutions are oriented to fire detection in outdoor forest environments. Still, there is an added problem in indoor environments when there is also a lack of communication and GNSS connectivity to send information about outside^9^. Another problem is the acquisition systems, which must allow operating under extreme environmental conditions (high temperatures and lack of visibility^9^.

In this line, Roldán-Gómez et al.^10^ suggested the use of autonomous navigation systems to provide information, thus no imposing static conditions indoors. This could help other scenarios such as airborne pathogens to monitor and control pandemics, allergies, etc.

Mitigation of fire risk and hazards require effective and accurate forecasting systems. Satellite-based solutions have been previously consider in the literature, such as the work of Fukuhara et al.^11^, who analyzed satellite images to detect hot spots (areas emitting high levels of radiation). Unfortunately, this method requires successive scans over the same area with the consequent excessive time consumption for analysis.

Smart Cities challenges are benefit considerably from the rising of Big Data information coming from IoT devices, improving monitoring and prevention processes. Nevertheless, this methods require visualization techniques as a key component of interpretation and development. The prevention of possible hazards (such as fires, leaks, etc.) , monitorization and resource optimization benefits considerably by means of visualization tools capable to provide insights to decision makers^12^. In this line, we propose a platform based on sensors and detectors (beacons) to improve their intelligence to classify the detected ambient ($\text {CO}_2$, humidity, temperature, toxicity, chemical agents, etc.) parameters and agents through predictive modeling. Depending on the rest of the parameters recorded (environment, space, dimensions, type of accident, etc.), the system will help to classify which agent is most likely to be detected. The first deep learning (DL) applications are due to Freeman et al.^13^, to predict air pollution time series using deep learning by means of recurrent neural network (RNN) with long short-term memory (LSTM). Other authors, such as Liu et al.^14^ proposed hybrid approaches combining ARIMAX models with numerical modelling to forecast air quality, Samal et al.^15^ who combined SARIMA and Prophet model for the air pollution levels of Bhubaneswar City and Shehhi and Kaya^16^ that explore time series machine learning models to characterize the non-stationary time series data and predict future values. Air quality models based in time series can be found in Zivot and Wang^17^, Rekhi et al.^18^ and Hepziba Lizzie and Senthil Kumar^19^. Other works based in Bayesian deep-learning were suggested by Han et al.^20^. A comparative of statistical and deep learning methods to forecast long-term pollution trends is described by Nath et al.^21^.

Classical estimation and prediction techniques of linear regression models will be used to analyze the series. The adequacy of dynamic regression models (such as ARIMA) in time series technology will be studied. Box and Jenkins^22^ proposed this type of model for stationary time series with linear autocorrelation. The main objective of developing them is to estimate and predict their behavior based on the past behavior of the same series in which regression variables will be used. The ARIMAX model (p d q) is a variant of the ARIMA model containing explanatory external variables to generate predictions. In general, it improves the quality of forecasts and allows you to manage variables in decision-making. The functional form is identical to that of ARIMA but with exogenous variables. In addition to the ARIMAX models, the following models will apply KNN (K-Nearest Neighbors), SVM (Support Vector Machine), and TBATS (Trigonometric seasonality, Box-Cox transformation, ARMA errors, Trend and Seasonal components). The method used in this paper will incorporate a time series adjustment model. The idea of predictive combination assumes that it is impossible to determine the underlying process of explaining the phenomenon through a single model, and each predictive model can use different types of information available for prediction.

Thus, we describe a platform to provide security, prevention, rescue, and evacuation services in indoor interventions without GNSS (Global Navigation Satellite System) connectivity and communications with the outside. Our main objective is the evolution of information monitored caused by the fire source and its evolution over time, before and during emergency personnel (firefighters) intervention, even when the fire is almost invisible. We use them in a timing series to acquire environmental parameters. We have used smoke machines with liquids that simulate smoke in a fire with high temperatures. However, an elevated fire was not simulated for the safety of the researchers. A combination of predictions made with different technologies can be the most accurate prediction. In addition to the many techniques used to specify individual predictions, the methods used to obtain combined predictions are also very diverse. Therefore, the choice of combined methods and combined individual predictions will depend on the preference of the researcher, the complexity of the technology used, and the cost and precision provided by the forecast. Related to the autonomy of the devices to monitor spaces, in many emergency scenarios, especially in indoor fire situations, the firefighters can involve compromised power infrastructure or limited resources. Our system will be designed specifically in order to address the need for a power-efficient solution to ensure continuous operation in resource-constrained environments.

Nevertheless, this technique provides the best prediction results collected from the diversity of each previously used fitted model. Therefore, our proposal would allow for reducing and minimizing the exposure to risk and possible accidents, offering added levels of visibility and information on the conditions of the environment that are not known. In the design and development of the proposed system, we have followed the recommendations and needs of the APTB (Professional Association of Fire Technicians) and the management of 112 Madrid, which have allowed us to carry out evaluations of our research in the training centers described in the Test Design section.

## Methods

### Architecture

**Figure 1:**
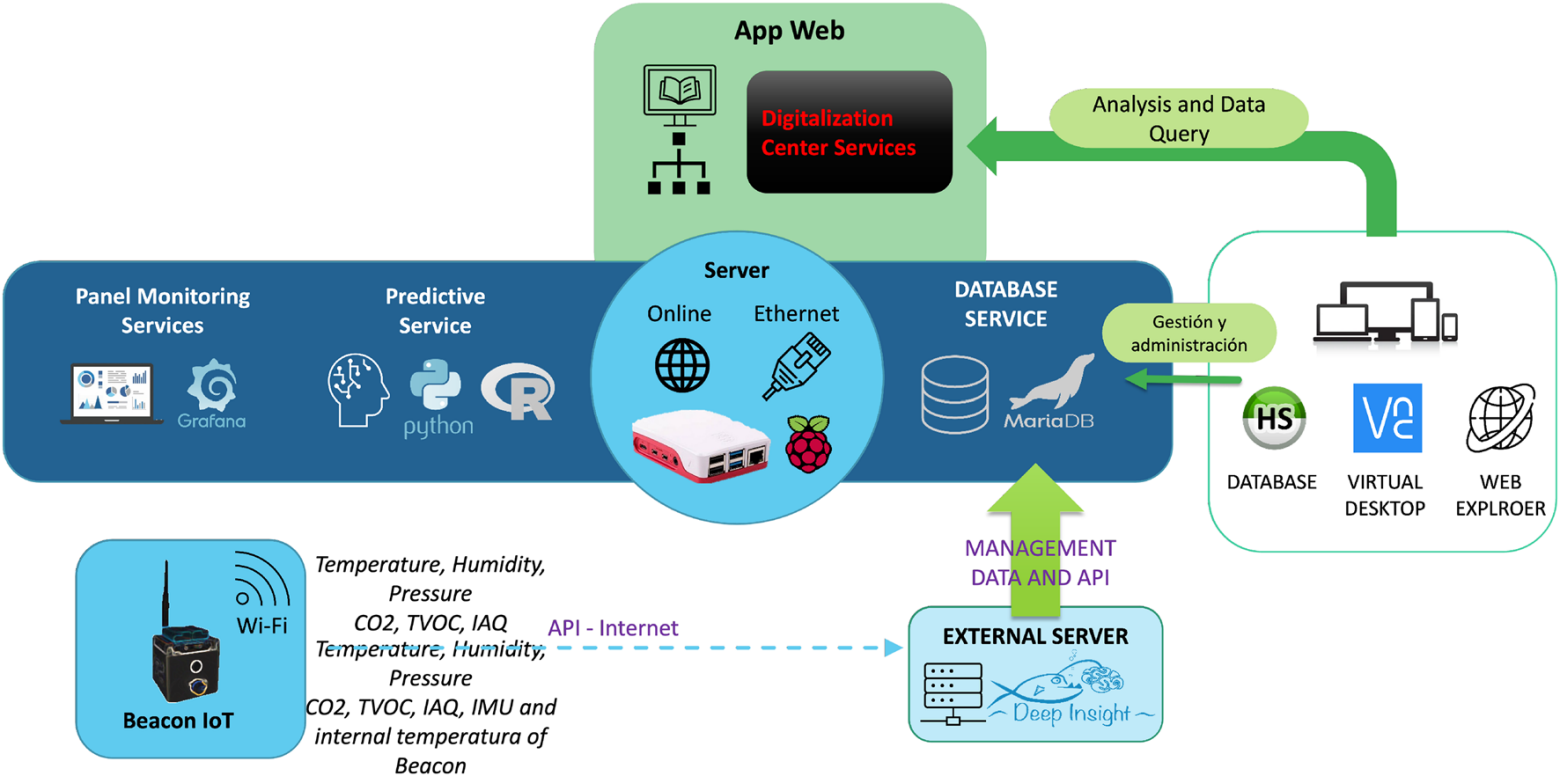
Architecture for the emergency contexts monitoring and predictive services.

**Figure 2:**
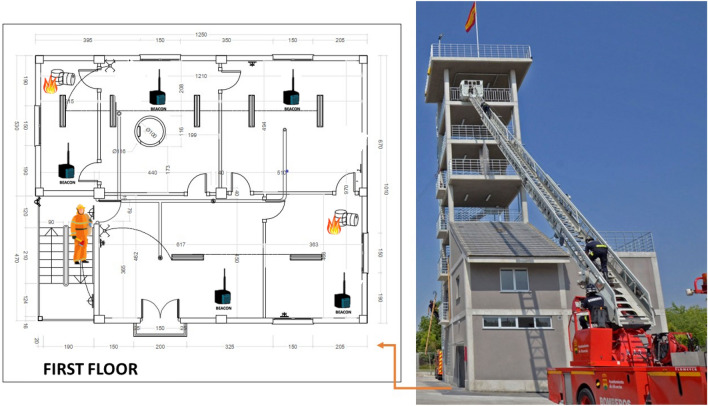
Example of deployment of beacons in a scenario with five rooms on the first floor of the Fire Tower in the Unified Security Center of Alcorcón. In this scenario, five beacons were used.

The approach proposed in this project (called *HelpResponder*) is to monitor the state of the emergency place to know environmental information and images in indoor spaces and the absence of GNSS communications (see Fig. 1). The study focused on the data acquisition, power consumption of beacons, and information modeling to predict emergency states. In fact, in this paper, we present a method based on the combination of predictions from different models to deal with the time series of the $\text {eCO}_2$ emissions collected by specific sensors. For the monitoring task of the quality of the environment, we have used a commercial detector used by Firefighters in the studied scenario. This device was used in the hands of firefighters. We made different measures to establish the relation among the rest parameters for this study. We chose the different sensors to be registered by the beacons in autonomously. After, we used autonomous monitoring without human interventions using beacons. For these beacons, we have used the next sensor, BME680, which includes the channels of temperature, humidity, pressure, siaq, air quality measurement, and the SGP30, which includes the $\text {eCO}_2$ and TVOC channels. To make better decisions to reduce fire risks and hazards, building an effective and accurate forecasting system is necessary. The intervention carried out by the first response team will be supported by an App adapted to emergencies, portable and multi-platform, which offers an interface for real-time monitoring through panels and information maps (dashboards). The deployment for this study is shown in Fig. 2. The following figure shows the pilot space used to evaluate the models and the prediction of the proposal presented in this article. It is a housing-type plant composed of 4 rooms connected by a door. Beacons have been installed in each of the rooms. These beacons were installed during the evaluation of the pilot project. This deployment has been used to evaluate the evolution of the environmental agents. In this space, different pieces of training have been carried out by the emergency services, training, small manipulated and controlled fires, etc. For this study, we have used smoke liquids, flammable liquids, to simulate gas leaks, fire from burning pans or candles and liquid spills. The data transmission platform installed on the beacons allows evaluation in a compartmentalized space, as shown in the figure, and in an open-plan garage-type space.

This system has been evaluated in confined spaces (indoor). We have used smoke machines with liquids that simulate smoke in a fire with high temperatures. However, an elevated fire was not simulated for the safety of the researchers. The Fire Tower of the Unified Security Center of Alcorcón (CUS) is dedicated to the practices of emergency personnel in the face of real interventions. Therefore, it is possible to emulate real dwellings. Moreover, tests have been carried out in the laboratories and facilities of the Rey Juan Carlos University.

#### Beacon network

**Figure 3:**
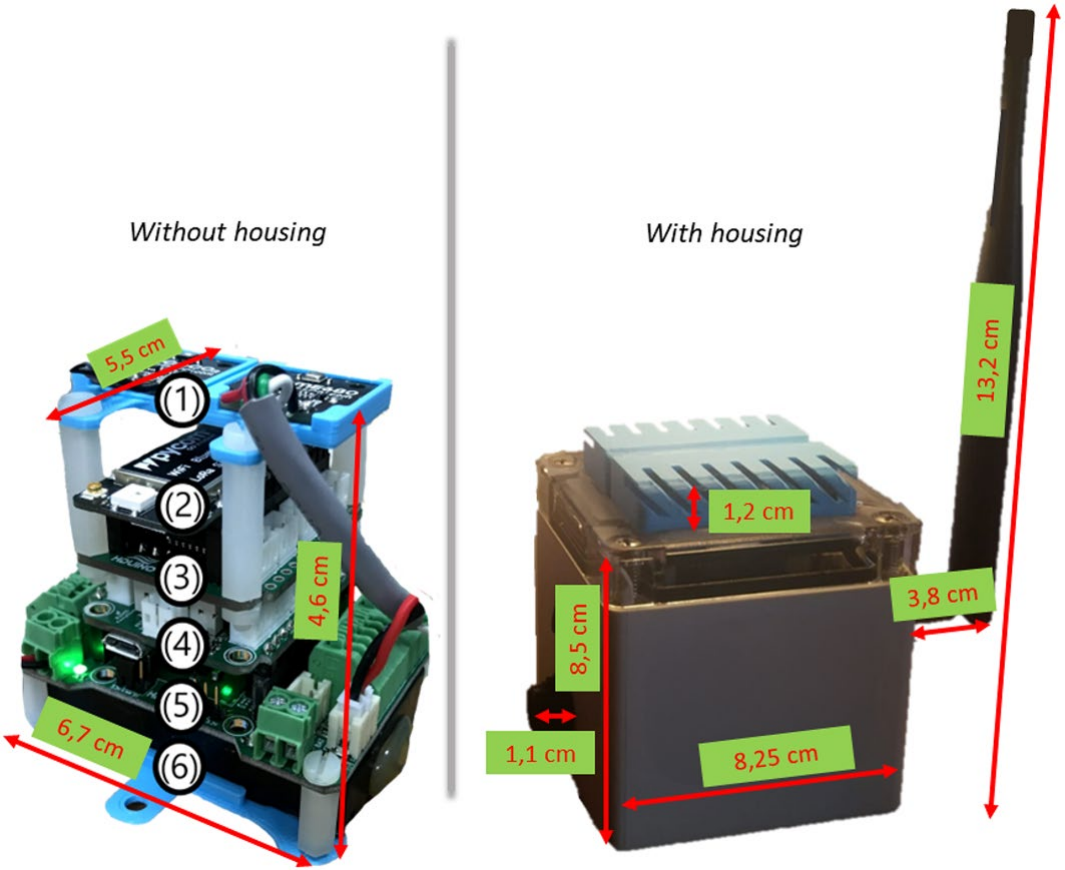
Module (2) Beaconing system. Deep Beacon—cutting.

It consists of multipurpose acquisition and control devices based on a Deep Beacon architecture. Depending on its use, it can go without a housing reaching a weight of 125 grams and measures of 5.5 (length) $\times$ 6.7 (width) $\times$ 4.6 (height) centimeters used, for example, in the case of the AGV (Autonomous Ground Vehicle) or with housing designed for hostile environments with an IP67 protection index reaching a total weight of the device of 358 grams and measures of 13.15 (length) $\times$ 8.25 (width) $\times$ 13.2 (height) centimeters used for example for deployment in interventions. In Fig. 3, we can see the measurements for without and with housing and or architecture, which is based on different levels or layers (see Table 1):

1. Sensing layer: here, we have the external sensors connected to the device, the SGP30, BME680, and LIS3DH.
2. Communication layer: includes a LoPy4 to manage wireless communications by WiFi and LoRa.
3. Storage layer: it has a MicroSD slot to store sensed data.
4. Acquisition layer: central monitoring module with the microcontroller responsible for data acquisition tasks. This belongs to the series of ultra-low-power 32-bit microcontrollers, based on the Arm®Cortex®-M0+ processor architecture. It has internal monitoring sensors for battery charging, temperature, and humidity of the box.
5. Power management layer: dedicated to controlling power and battery charging.
6. Autonomy layer: the 3.7 V battery.

**Table 1:** Levels for the hardware into the beacons.

	Description
Sensing level	GP30, BME680 and LIS3DH
Communication layers	LoPy4 for the management of Lora and WiFi
Storage layer	Storage
Acquisition layer	Ultra low-power 32-bit microcontrollers series, based on the Armtextregistered Cortextextregistered-M0+ processor architecture
Power and Autonomy Management laser	Battery Charger with 3.7 battery

The function of this device is to know the status of the intervention, which areas of exit and entry are safe, how the fire evolves, and the detection of fire hotspots. To perform these functions, this device has several operating modes, depending on the kind of intervention, one or the other can be used:

- Continuous transmission mode: in this mode, we are working in the maximum power mode, always on and sending data wirelessly via WiFi or LoRa at the maximum transmission rate (1 transmission every 10 s).
- Power saving mode: in this mode, data is sent wirelessly by WiFi or LoRa when desired, turning off the device between shipments to save the maximum battery, looking for greater versatility and range of system use. Pre-programmed modes of shipping per minute: 4 shipments per minute, two shipments per minute, one shipment per minute, and one shipment every 10 min.
- Data-logger mode: in this mode, you do not have the wireless send module active, only the microSD data storage mode.

Suppose the system detects a sudden rise in $\text {CO}_2$ concentration (a fire event happens suddenly). In that case, the software of the beacons starts to monitor to store the maximum possible data to learn from the environment. In future research, we will modify the software of the server and beacons to improve this measurement and economize the beacon’s battery.

#### Interoperable and holistic data models

Data received from sensors is made available to services that need it through data exchange APIs, thus promoting open data principles. Data models follow the recommendations and structures proposed in the knowledge layer of the UNE 178104:2017 standard. The stored data are structured in various containers according to their purpose. Thus, there are repositories for:

- Historical data stores time-series measurements coming directly from sensors. It serves to maintain the persistence of all data sets. It uses a NoSQL database (Cassandra), which is open source-oriented to BIG Data and IoT. It serves us as a gateway for any type of data as it is not structured, as a backup to recover the dataset if necessary, and as free-up space in the main containers.
- Structured data. This container is structured/relational data, stored in a relational database (MySQL/MariaDB), used to organize and manage information related to the devices that emit data (location, type of sensors, status, models, etc.) as well as to store the values of the readings.

#### Data exchange API

**Figure 4:**
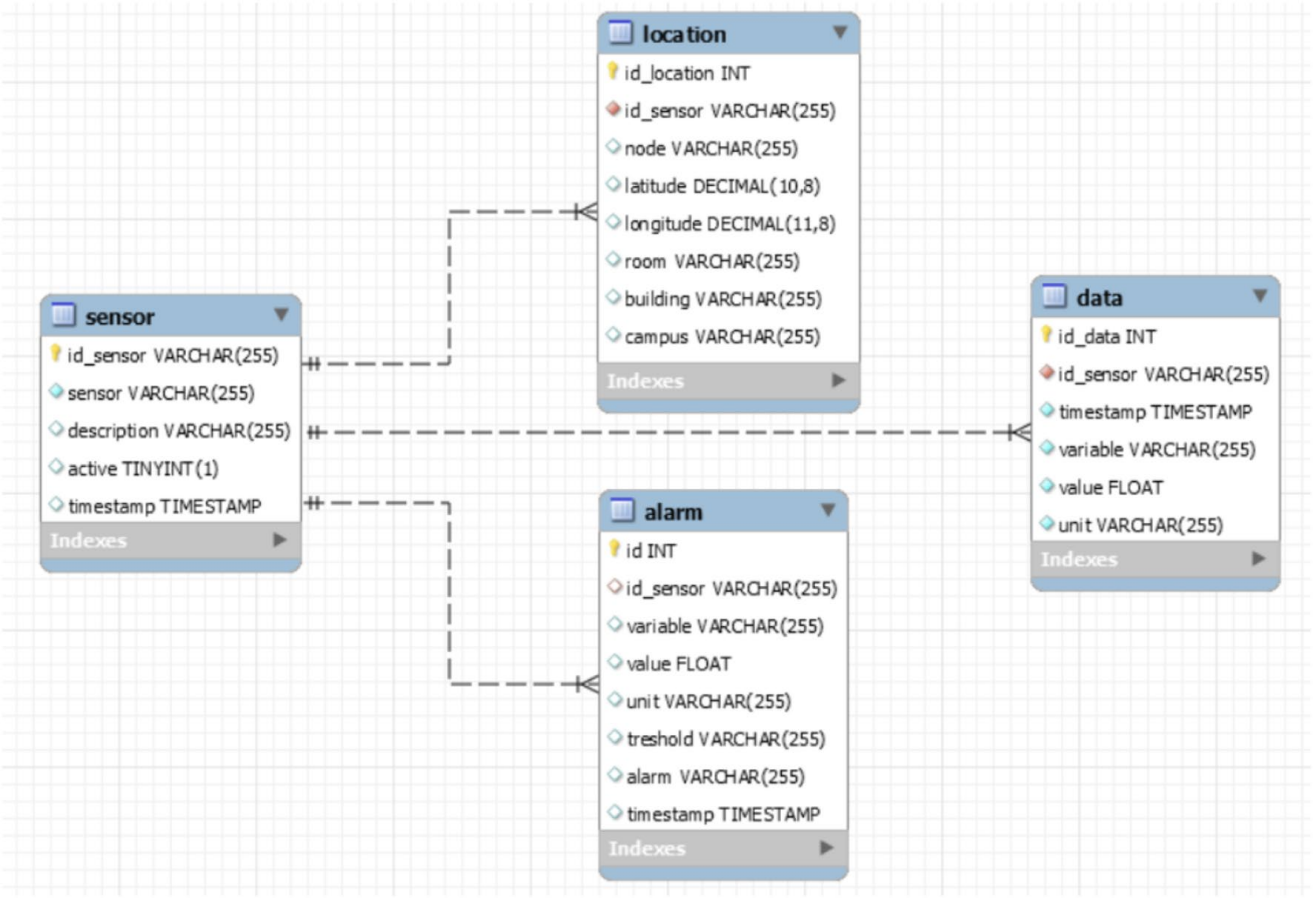
Database schema for storing sensor data in the monitoring process.

The stored data are shared through APIs, which provide a gateway service between different services or languages based on JSON documents. The mechanisms used comply with the interoperability layer of the UNE 178104:2017 standard. These APIs facilitate the exchange of information between services and also enable interoperability. The registered data from beacons have been stored in a database using MySQL format (see Fig.  4).

#### Prediction models

To make better decisions to reduce fire risks and hazards, building an effective and accurate forecasting system is necessary. A combination of forecasts from different models is used to predict the $\text {eCO}_2$ emissions collected by the sensors. The models studied are:

- ARIMAX models. These are ARIMA models that incorporate exogenous variables into the adjustment. The Arima method^23^, or Box-Jenkins method, focuses on the autocorrelation between observations, describing each value as a linear function of previous data and errors due to chance, and may include a cyclical or seasonal component.
- The K-nearest Neighbors method (KNN)^24^, which is a non-parametric method that selects the k closest observations according to a distance metric. Once the closest neighbors have been selected, an aggregation measure is calculated to obtain the forecast.
- Support vector machines (SVM): a learning method used for time series prediction. The parameter estimation is carried out by minimizing a risk function where the empirical error between the model and the data is measured and a regularization component that depends only on the weights. The SVM function of the R package e1071^25^ trains a support vector machine. This function can be used to perform general classifications and regressions, as well as to estimate density. A kernels polynomial is used, and explanatory variables with autoregressive structure and dummy variables to capture seasonality are added. The incorporation of this type of explanatory variables improves significantly the SVM prediction procedure.
- Trigonometric seasonality, Box-Cox transformation, ARMA errors, Trend, and Seasonal components (TBATS), which was introduced by De Livera et al.^26^ to forecast time series with complex seasonal patterns using exponential smoothing.

Three prediction combination models are chosen from the literature: the arithmetic mean, a weighted mean based on variances and covariances (it calculates the weights of a combination of forecasts using^27^’s approach and produces forecasts for the test set if provided), and contraint least squares, which calculates forecast combination weights using restricted least squares (CLS) regression.^28–30^.

Rolling window tests are carried out for the prediction procedure within the sample. To perform a rolling window analysis, the data is split into an estimation sample and a prediction sample. The model is fitted using the estimation sample, and k-step-ahead predictions are made for the prediction sample. k-step-ahead prediction errors are computed because the data used to make the predictions is observed. This process is repeated until no more k-step predictions can be made^31^. The Mean Absolute Error (MAE) and Mean Squared Error (MSE) prediction error measures are used to evaluate the performance of the models. In situations where multiple sensor parameters are involved, hyperparameter tuning for each candidate model using a single time series to minimize the error metric can become more complex. To evaluate the performance of ARIMA, SVM, TBATS, and KNN, their parameters are recalculated in each iteration of the validation process by fitting them to the current window train data (i.e. in each iteration new SVM and KNN models are trained and ARIMA and TBATS models fitted). The process was repeated for each window to choose the model that minimized the error metric over all the windows.

#### Web interface/data visualization application

HTML5, CSS3, and JavaScript have been used for web design. This App Web allows accessing the different dashboards or data visualization panels. A web interface has been used. To access the different dashboards or data visualization panels, the main page has been created where they are all listed (graphically and with text). Currently, we have deployed the following options or kinds of Dashboards:

1. Dashboard Monitoring: it has information related to the reading of all the sensors connected to the selected beacon.
2. Alarm Dashboard: shows the status of the sensor readings and if any of these have exceeded the alarm threshold.
3. Dashboard vision: it has the information of all the sensors of the beacon and the visualization of thermal images and fire detection in real-time.
4. Dashboard Tests: This section lists the different dashboards intended for monitoring specific variables important for certain tests that are being carried out.

**Figure 5:**
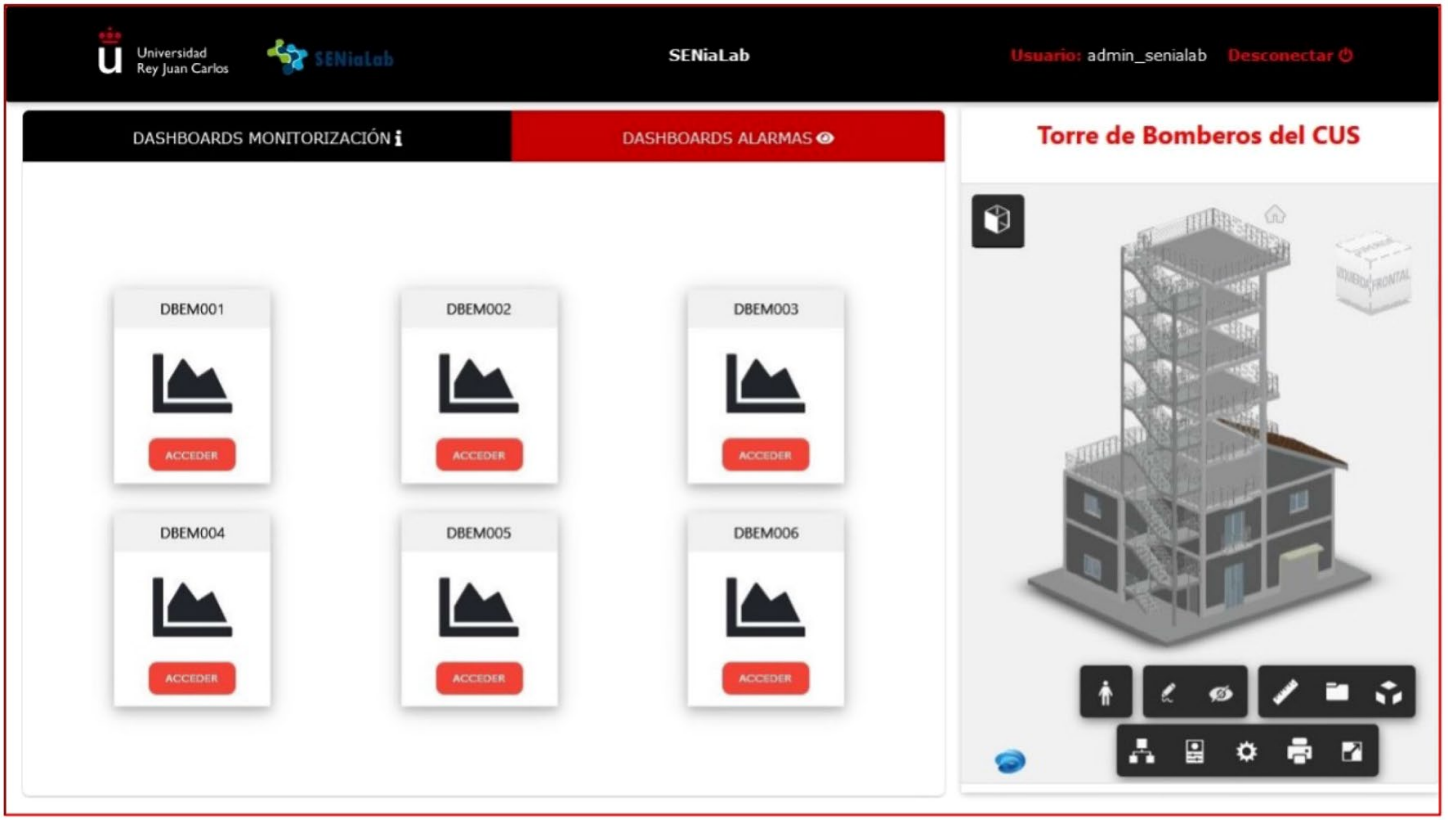
Module 3 (Windows to choose the Monitoring Application Services).

**Figure 6:**
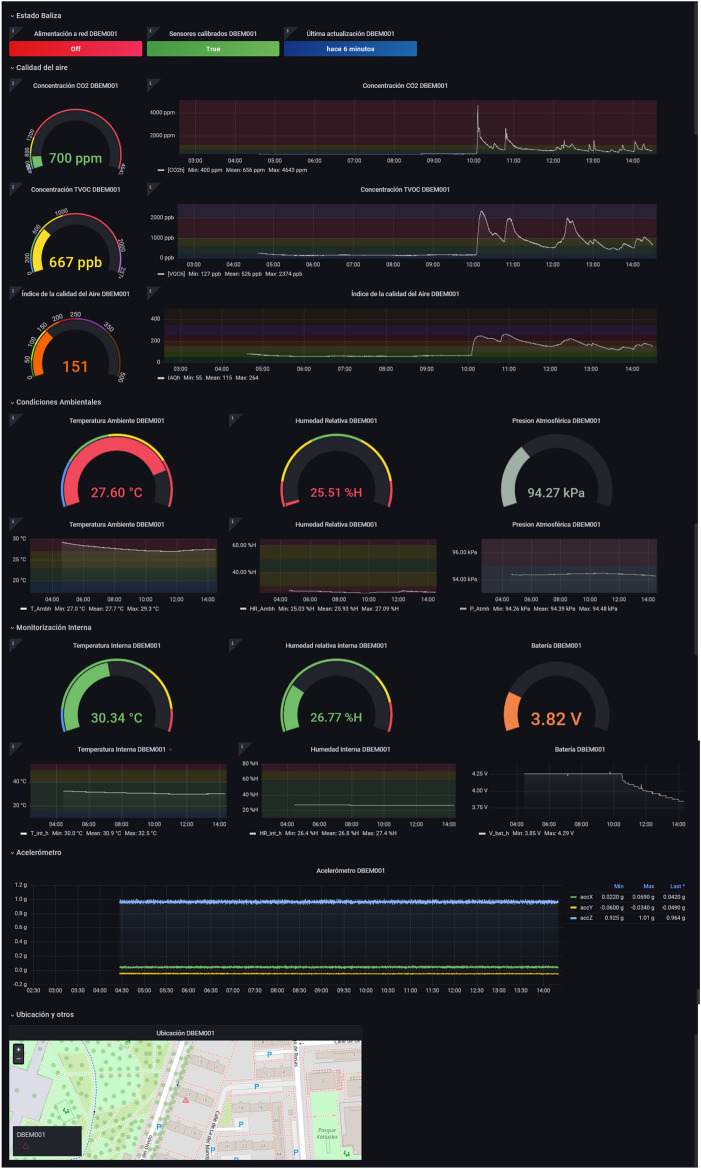
Module 3 (panel control of the monitoring application).

Figures 5 and 6 show an example of a real application where we can observe different dashboards accessible from a list or section created especially for the *HelpResponder* project. Figure 5, it is shown the interface to choose the type of information for a query; in Fig. 6, the dashboard with the different choices is shown. The final user can see the different data from the monitoring and choose the Beacon or place.

#### Server

All necessary services are installed on a server based on an Ubuntu 18.04 LTS (Long-Time Service) machine. In addition, the web interface for the visualization of the data collected by the fire detection system and the beaconing system runs under an Apache HTTP Server Version 2.4 web server. Finally, the database selected to store all received data is NoSQL, specifically Apache Cassandra 3.11.

Also, there is a server for executing data postprocessing programs for alarms, mailings, filters, etc., based on Jupyter Notebooks.

## Results

### Autonomy of the Beacon network for monitoring and prediction system

The purpose of the tests carried out for the beacon network is to study and know the evolution of autonomy depending on the transmission rate. From these results, we can adapt the shipping rate according to the needs of each type of intervention where the beacon system is deployed. Therefore, this system has been validated with two different tests.

#### Test 1

The first test consisted of transmitting data 24 h a day for 24 days with the beacons connected to the electricity grid in different environments, including the CUS, where the beacons were placed on the same floor and in different rooms. In addition, three beacons were placed issuing at three different transmission rates, 10 s, 1 min, and 10 min. Most of the time, they were in a simulated environment, and on specific days tests were carried out with smoke and real fire a few meters from the beacons.

After all the tests of this type carried out, we conclude that the beaconing system had been able to send data in all the programmed sending frequencies that have been discussed in previous sections, 6 times per minute, 4 times per minute, 2 times per minute and 1 time per minute and 1 time every 10 minutes, both in simulated environments and in real environments with hot smoke and fire. The time frequencies have been considered sufficient to respond to the monitoring of relevant emergency data^4,5^.

#### Test 2

**Figure 7:**
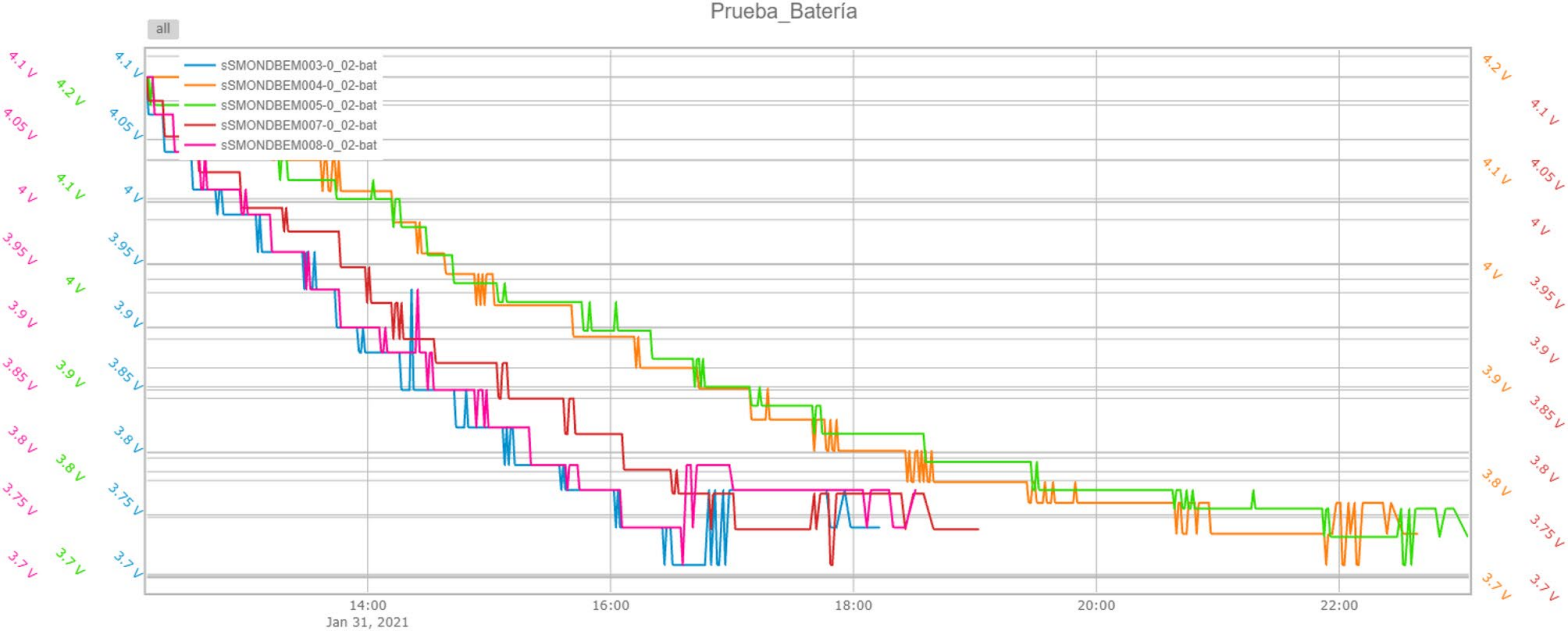
Autonomy graph of the beaconing system.

The second test is oriented to know the autonomy we can achieve with this system. This network can be connected to electricity to send uninterrupted environmental variables or connected to the battery that incorporates 3.7 V and 2000 mAh, achieving autonomy with the node at its maximum operation of up to 11 h. In Fig. 7, it can be seen the battery life of an example of the beacon system composed of 5 nodes. As mentioned in previous sections, each beacon consists of several layers, the ones with the highest consumption are the acquisition layer and the communication layer:

1. The acquisition layer must be working at its best at all times, as it is continuously performing air quality processing, sensor calibration, etc., so it cannot be turned off. This layer consumes 52 mA.
2. On the other hand, the communication layer, with the WiFi or LoRa on and transmitting, consumes 113 mA

Based on the obtained results, it can be concluded that the total consumption of each beacon is 165 mA, which, as we have mentioned, can be in battery mode or connected to the electricity grid without consuming the battery. Although the autonomy achieved of about 11 h maximum for transmissions every 10 s seems sufficient for an intervention, the possibility of battery savings arises to increase its range of use and scalability. This can be reached by “sleeping” between data transmissions and the communication layer. In this way, we sometimes manage to reduce the consumption of the system to 52 mA during those periods.

To estimate the battery life, theoretical calculations will be made, followed by real tests. For our case with a 3.7 V battery and 2000 mAh, we get a power:

1a $$\begin{aligned}{} & {} P=Voltage*Charge, \end{aligned}$$

1b $$\begin{aligned}{} & {} P=3.7 V*2000\,\, mAh = 7400\,\, mWh, \end{aligned}$$

The board has DC–DC converters to pass the battery voltage to 5 V, assuming that the losses are negligible. Since this type of converter, unlike the regulators, have a high performance, the capacity of the battery of our system is achieved by clearing the Eq. (1a):

2 $$\begin{aligned} I=\frac{7400\,\, mWh}{5V}=1480 \,\,mAh, \end{aligned}$$

The actual tests to be performed consist of 7 tests with 5 beacons for each data transmission frequency, taking data related to the initial/end voltages, start/end day, and start/end time. In this way, we will carry out an in-depth statistical analysis to select the highest transmission rate in the future.

It should be noted that each beacon has been configured to charge the battery up to a certain voltage to make a broader study on the autonomy later. So it is the most representative of reality the beacon ”DBEM005” that is charged completely.

Let’s consider a frequency of 10 s of dispatching data, i.e. 6 times per minute, then the system consumption is 165 mA. Applying Eq. (3a), the theoretical autonomy in hours is given in Eq. (3b).

3a $$\begin{aligned}{} & {} Autonomy=\frac{Capacity}{Intensity}, \end{aligned}$$

3b $$\begin{aligned}{} & {} Autonomy=\frac{1480 \,\,mAh}{165\,\, mA}=8.9\text { hours} = 8\text { hours and }58\text { minutes}, \end{aligned}$$

The real autonomy data give us the following results (see Table 2):

**Table 2:** Real autonomy results for 6 transmissions per minute.

	Beacon				
	DBEM003	DBEM004	DBEM005	DBEM007	DBEM008
VO [V]	4.07	4.2	4.23	4.1	4.1
Vf [V]	3.74	3.74	3.74	3.74	3.74
Autonomy	5:54	10:30	10:46	6:38	6:24

Upon comparison of the theoretical results obtained and described above with the final result, it is evident that the estimation is accurate. Furthermore, due to the software configuration that enhances autonomy, we were able to observe additional details. For instance, we noticed that the beacon labeled ‘DBEM005’ was higher than the estimated value by + 1$^\prime$48${^\prime} {^\prime}$.

In the case of 4 shipments per minute, we have verified that we can “sleep” the communication layer for 20 s. Consumption is as follows: First, we will calculate the percentage of time that the beacon is in normal mode and “sleep” mode

4a $$\begin{aligned}{} & {} \%t_{1}=\frac{t_{1}}{t{Total}}, \end{aligned}$$

4b $$\begin{aligned}{} & {} \%t_{Normal}=\frac{40 \text {seconds}}{60 \text {seconds}}=0.67, \end{aligned}$$

4c $$\begin{aligned}{} & {} \%t_{Sleep}=\frac{20 \text {seconds}}{60 \text {seconds}}=0.33, \end{aligned}$$

Then, we can calculate the average consumption from the data:

5a $$\begin{aligned}{} & {} I_{average}=\%t_{1}*I_{1}+\%t_{2}*I_{2}, \end{aligned}$$

5b $$\begin{aligned}{} & {} I_{average}=0.67*165\,\,mA+0.33*52\,\,mA=127.33\,\,mA, \end{aligned}$$

Having, therefore, the autonomy of:

6 $$\begin{aligned} Autonomy=\frac{1480\,\,mAh}{127.33}=11.62 \text { hours}=11 \text { hours and }37 \text { minutes}, \end{aligned}$$

The real autonomy data give us the following results, which are an average of the 7 days of duration of the test (see Table  3).

**Table 3:** Real autonomy results for 4 transmissions per minute.

	Beacon				
	DBEM003	DBEM004	DBEM005	DBEM007	DBEM008
VO [V]	4.07	4.2	4.23	4.1	4.1
Vf [V]	3.74	3.74	3.74	3.74	3.74
Autonomy	6:44	12:05	12:31	7:33	7:18

Table 4 presents the theoretical (applying Eqs. (4a), (5a) and (3b)) and observed Autonomy for each beacon considering different shipments frequencies.

**Table 4:** Real autonomy results for different transmissions per minute.

	Beacon						
	Number of transmissions	Theoretical	DBEM003	DBEM004	DBEM005	DBEM007	DBEM008
VO[V]			4.07	4.2	4.23	4.1	4.1
Vf[V]			3.74	3.74	3.74	3.74	3.74
Autonomy	4	8:58	6:44	12:05	12:31	7:33	7:18
Autonomy	2	16:31	8:54	16:05	16:59	10:09	9:17
Autonomy	1	20:53	10:18	18:38	19:43	11:30	10:37

As observed, the theoretical calculations help us approximate the autonomy that we can achieve since we achieve results relatively close to reality. The autonomy of the other transmission modes has also been evaluated. We have detected a flat tendency not to achieve longer battery life from 1 send per minute due to the programming of the device. The communication layer restarts every minute to clean memory for safety and calibrate its internal clock. This limits us to turn it on at least once a minute, greatly disappearing the battery saving if we want to transmit at a rate less than 1 time per minute.

Therefore, the maximum time that the beacons can work on in an intervention is about 20 h, time in principle more than enough for intervention.

All these calculations and tests have also been designed to create statistical models that allow us to select the best transmission rate at all times based on the evolution of the variables sensed.

From these two tests related to the beaconing system, we can make it clear that we can deploy the system and monitor both in the long and short term in any of the modes of transmission described and in hostile environments with fire and smoke. In addition, it is of great interest to give the possibility of greater autonomy for the necessary moments.

We aim to emphasize the importance of having a battery-efficient system in emergency situations. Many emergency scenarios, especially in indoor fire situations, can involve compromised power infrastructure or limited resources. Our system has a design that specifically addresses the need for a power-efficient solution to ensure continuous operation in resource-constrained environments.

Finally, it should be noted that the nodes have a WiFi connection to upload the data taken by the sensors to the cloud. In addition, they have an emergency mode, which is activated when they cannot connect to a WiFi network, activating LoRa wireless connectivity to send the data to an external station (EVA) that will upload the data to the cloud.

### Evaluation of the combination of predictions models

Given the complexity of the data, it seems reasonable to assume that the underlying process cannot be identified by a single model. Different models may capture different aspects of the information, which leads to different predictions. For this reason, it is proposed to use a combination of the predictions from different models.

**Figure 8:**
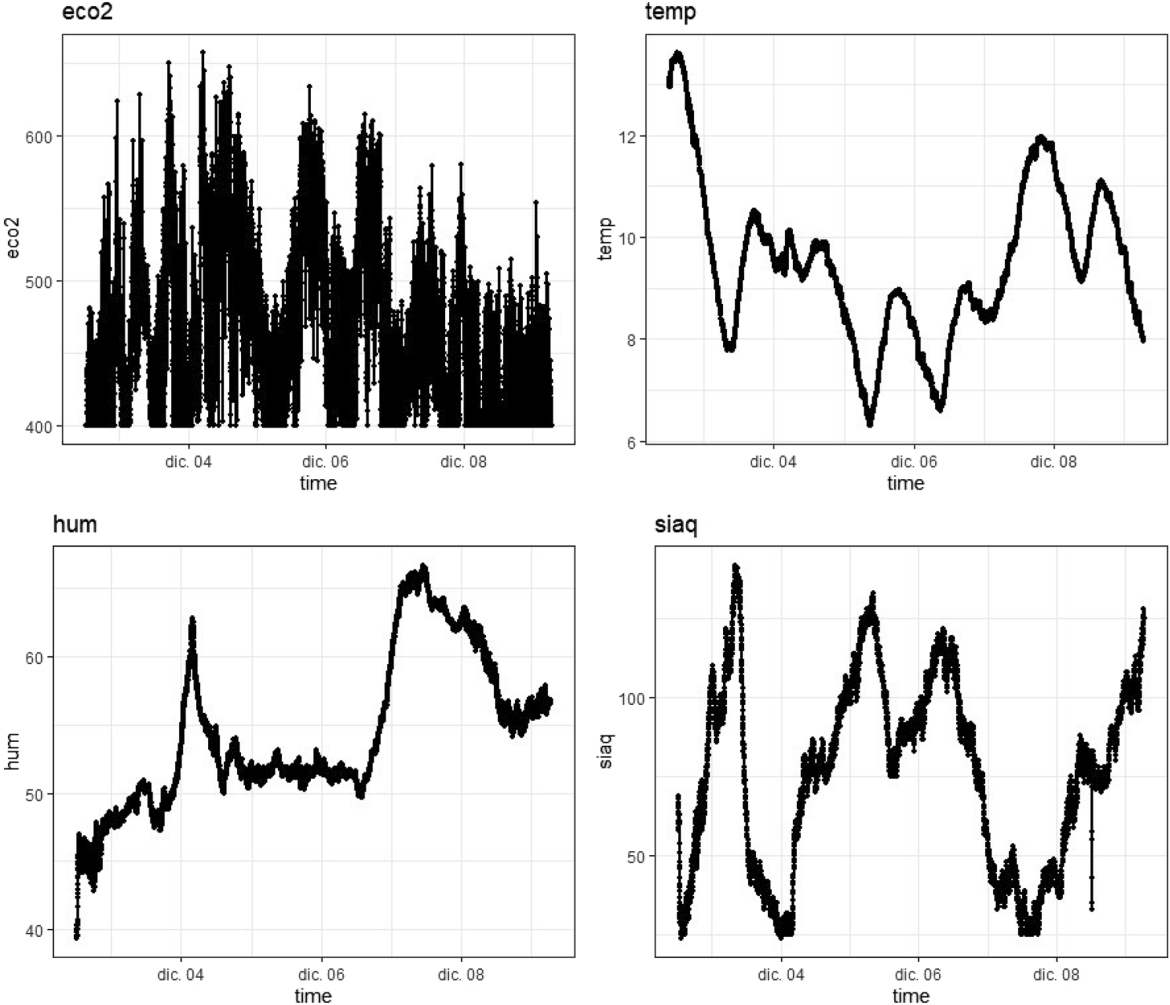
Time series $\text {eCO}_2$, temperature (temp), humidity (hum) and pressure 03-siaq (siaq).

The study is carried out with data collected in the Alcorcón fire tower with the DBEM002-0 beacon. The aim is to estimate the model that best predicts $\text {eCO}_2$ values, using the variables: temperature (temp), humidity (hum), and air quality (siaq). Figure 8 shows the data collected for each of the variables. Information has been extracted from the following sensors:

- “sWEA” (BME680) includes the channels of temperature, humidity, pressure 03-siaq, air quality measurement.
- “sAQU” (SGP30) includes the $\text {eCO}_2$ and TVOC channels.

**Figure 9:**
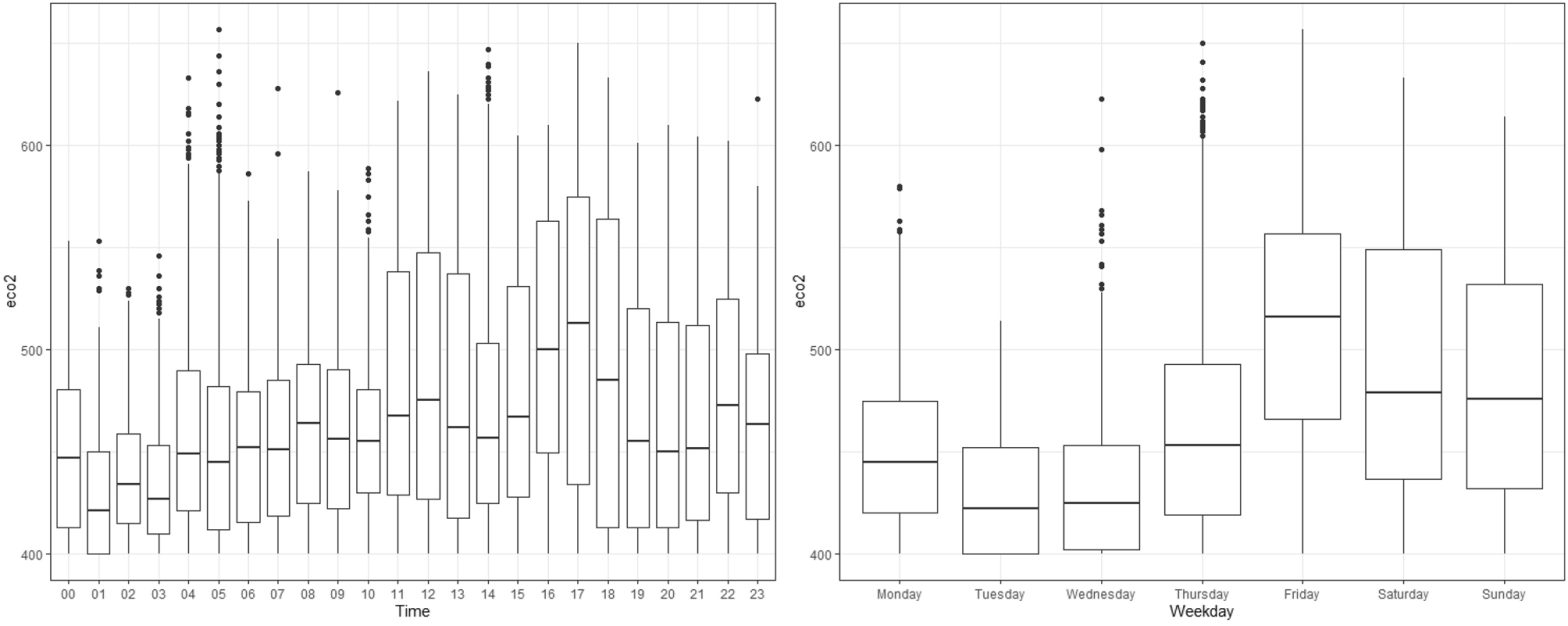
Hourly (left) and Daily (right) boxplots for $\text {eCO}_2$.

Data is collected every minute from 12/2/2020 at 12:10 to 12/9/2020 at 6:38. Figure 9 shows how the response variable of interest ($\text {eCO}_2$) varies by the hour and day of the week.

**Figure 10:**
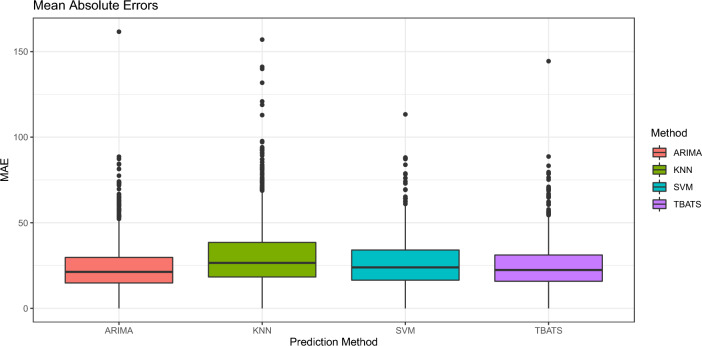
MAE boxplots obtained from the Rolling Window for each Model.

There appear to be differences in the concentration of $\text {eCO}_2$ throughout the day. Higher values of concentration are observed in the early morning hours (from 00 to 10), and more dispersion from 11 to 23, with higher values. The same comparison was made by day of the week, and changes in concentration were observed between the days of the week (more concentrated with lower values) and the weekend (more dispersed and higher values). The Rolling Window is chosen to be 120 min, from which 10 min ahead are predicted. In each iteration, the window advances 10 min, so there are a total of 962 windows. The errors associated with these predictions are shown in Fig. 10 and Table 5.

**Table 5:** Prediction results at 10 min.

	ARIMA	KNN	SVM	TBTS
MAE	24.26	31.16	26.43	24.97
MSE	1108.22	1767.04	1263.74	1113.59

Related to the time of the prediction, our objective is not to predict long-term conditions in such scenarios but rather to provide timely insights that are aligned with the operational context of firefighting interventions. Therefore, we can conclude that within this 10-min interval, our system can contribute valuable insights that aid in informed decision-making and enhance the overall effectiveness of firefighting operations.

It should be noted that the ARIMA and SVM models include regressive variables, while KNN and TBATS are based exclusively on lagged values of the $\text {eCO}_2$ variable. In general, considering that the $\text {eCO}_2$ values range between 400 and 657, the errors are relatively small (the mean $\text {eCO}_2$ value is 466.9, with a standard deviation of 57.55).

**Figure 11:**
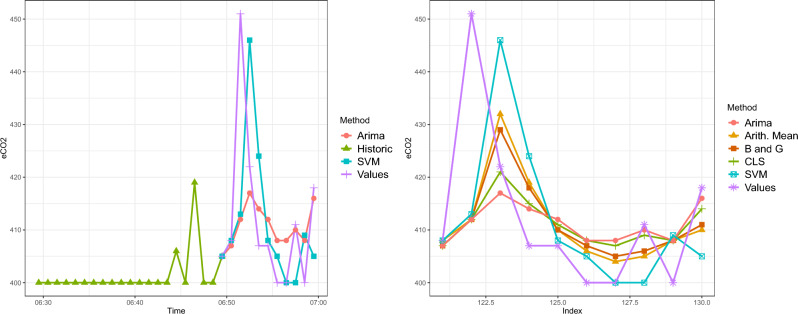
Example 1: last 20 min of historic with 10-min forecast (left) and predictions with combinations (right).

On the other hand, the irregular behavior of the variable provokes particular cases more difficult to be predicted. For example, peaks in the time series not previously observed, are unable to be predicted by time series models; however, their approximation could be tight-fitted. Figure 11 presents an example of parametric models in which only the last 20 min of the history and their corresponding adjustments and predictions are drawn (although 120 min of history were used).

**Table 6:** Weights of the combination of predictions for ARIMA and SVM models for Example 1.

	ARIMA	SVM
Arithmetic mean	0.5	0.5
Bates and Granger	0.58	0.41
Constrained Least Squares	0.85	0.14

The combination trains the weights based on the models’ predictions and their real values to reduce the error. For a given set of predictions, computed weights allow calculating predictions in a larger horizon. In this case, as we have 10-min predictions, 5 are used to train the weights, and 5 are used to calculate the predictions and corresponding MAE. The results shown in Fig. 11, are chosen to illustrate the weights (see Table 6). It can be seen that both methods give more weight to the ARIMA models than to the SVM model. Next, we partition the dataset into a training and test set. MAE and MSE can be calculated for both the training and test set of predictions, with each model and each proposed method of combination. The first 5 predictions (train) and the last 5 predictions (test) are considered separately, see Table 7, and finally, the whole data set is used, see Table 8, where it can be seen that the CLS method reduces the total error.

**Table 7:** MAE and MSE of the different methods of a combination of predictions for Test and Train when the first 5 predictions (train) and the last 5 predictions (test) are considered (Example 1).

	Arima		SVM		Arithmetic Mean		Bates Granger		Constrained Least-Squares	
	Train	Test	Train	Test	Train	Test	Train	Test	Train	Test
MAE	11.4	5.4	16	7.6	12.7	6.5	12.12	6.31	10.69	5.71
MSE	324.2	39.4	462	79.2	345.15	44.55	334.55	41.51	320.39	37.86

**Table 8:** MAE and MSE of the different models and their combinations of predictions for the total set (Example 1).

	ARIMA	SVM	Arithmetic Mean	Bates and Granger	Constrained Least Squares
MAE	8.4	11.8	9.6	9.21	8.2
MSE	181.8	270.6	194.85	188.03	179.13

**Figure 12:**
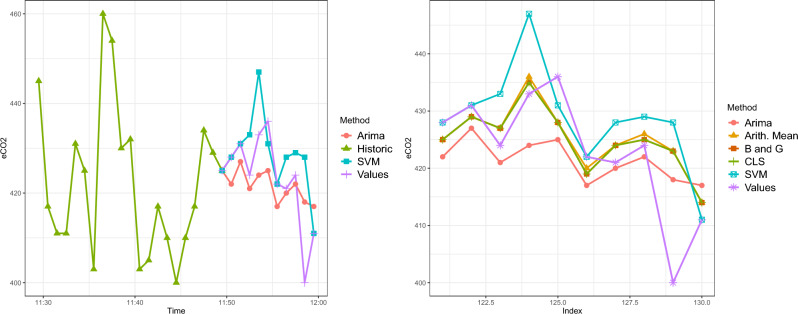
Example 2: last 20 min of historic with 10-minute forecast (left) and predictions with combinations (right).

Another example is shown in Fig. 12, making use of the weights for the different combinations shown in Table 9.

**Table 9:** Weights of the combination of predictions for ARIMA and SVM models for Example 2.

	ARIMA	SVM
Arithmetic Mean	0.5	0.5
Bates and Granger	0.5345	0.4655
Constrained Least Squares	0.5256	0.4744

It can be seen that both methods give slightly more weight to ARIMA than to SVM, again. MAE and MSE for both the training and test set of predictions, with each model and each proposed method of combination, are shown in Table 10. The overall MAEs and MSEs are summarised in Table 11.

**Table 10:** MAE and MSE of the different methods of a combination of predictions for Test and Train when the first 5 predictions (train) and the last 5 predictions (test) are considered (Example 2).

	Arima		SVM		Arithmetic Mean		Bates Granger		Constrained Least-Squares	
	Train	Test	Train	Test	Train	Test	Train	Test	Train	Test
MAE	6.6	6.4	5.6	8	3.7	6.6	3.57	6.5	3.6	6.5
MSE	52.6	78	6.4	171.6	18.45	111.1	18.36	107.93	18.35	108.73

**Table 11:** MAE and MSE of the different models and their combinations of predictions for the total set (Example 2.

	ARIMA	SVM	Arithmetic Mean	Bates and Granger	Constrained Least Squares
MAE	6.5	6.8	5.15	5.04	5.07
MSE	65.3	116	64.8	63.15	63.54

In most of the cases studied (here, only two are shown), the smallest errors are obtained when using the combination weights obtained by Constrained Least Squares. Moreover, the Test Data (further away) shows a smaller error with CLS, which would be the general recommendation for this type of data.

## Discussion

This paper is integrated into the project *HelpResponder*. The main objective was to offer a support system that allows monitoring to prevent interior space risks in an emergency context, such as those derived from fire due to high temperatures without visibility. By visibility, we mean that the environment’s visibility is compromised due to a smoke emergency. For this reason, smoke machines have been used with liquids that simulate smoke in a fire with high temperatures. However, an elevated fire was not simulated for the safety of the researchers. Therefore, a fire was simulated with a liquid that emits fire for 30–50 s and smoke using a smoke machine with smoke liquid. In this regard, we conducted a study to determine the model that best predicts measured $\text {CO}_2$ values using temperature, humidity, and air quality data from fire tower sensors. We have applied ARIMAX, KNN, SVM, and TBATS to make the adjustments and model these variables. In addition, an improvement has been incorporated into the SVM model. This consists of the inclusion of explanatory variables for the temporal structure. While the main aim was to create an explanatory model capable of identifying and measuring the contribution of the factors interacting with the dependent variables under study, future research may include the use of machine learning methods (neural networks), see, for example^31^.

The proposed system offers a small-scale prediction system that can save batteries and be integrated into a system accessible to firefighters to evaluate the route and the best operational tactics. The system is designed to provide monitoring and prediction over a given period of time (i.e. 10 min interval), making it scalable for longer intervals. Furthermore, the system is designed to be a stand-alone system that does not require connection to the tower’s control system. It allows access to data and the environment through a web application, communication with the advanced command post, and the ability to log events to learn from emergency situations. The prediction models have been tested and validated in real environments.

This system has been tested and validated in different situations for a real hostile environment (inside a building similar to a home), in a space with controlled situations, with a real fire controlled by firefighters, making real emergencies. The objective was to predict the risk before the fire is higher and dangerous for human persons, even the firefighters. With this prediction model, the risk could be minimized. For the evaluation, the beacons were deployed, and a web application was developed. The data visualisation on the web allowed monitoring, controlling, and supporting the emergency team in these high-risk situations, especially due to the common conditions of low visibility generated by the fire. Our system has the potential to provide valuable information in an emergency intervention, and its use would affect firefighter response times, firefighter operation times, and the avoidance of potential accidents, which could help save lives.

As a result of the study, we propose the combination of multiple time series models to predict $\text {eCO}_2$. Combining forecasts from conceptually different models effectively reduces prediction errors and thus provides higher accuracy. The proposed set is constructed with four well-known forecasting models, and empirical findings show that the proposed technique outperforms each model.

In fact, in recent decades, statistical machine-learning techniques have greatly contributed to the development of data-driven forecasting systems that offer cost-effective solutions and improved performance. Meanwhile, ARIMA is one of the most well-known linear statistical models for time series forecasting. In this paper, we present a hybrid approach using linear and nonlinear models.

The best combination method for this data considered is the weighted mean with weights based on Constraint Least Squares. The weights are, in general, almost equal, favoring the ARIMA model together with the proposed improvement in the SVM model. In addition to all this, we will continue to include new systems for acquisition and decision-making in real-time. Currently, we are developing statistical algorithms for the prediction of dispatch rates, focus prediction, guided positioning algorithms, and the acquisition of physiological variables by the emergency team to support them in their interventions.

To sum up, according to the results shown, the fire detection system can operate with acceptable $\text {eCO}_2$ predictions to detect real outbreaks, and the implemented beacon network works reliably and in real-time, being able to modify its sending rate according to the autonomy needs of the intervention.

While existing systems may offer some degree of integration, our system’s contribution lies in its holistic approach to real-time monitoring, prediction, and tactical evaluation. Specifically addressing your question on route evaluation, our system provides real-time data on environmental conditions, such as temperature, humidity, and air quality, along with the predictive insights on $\text {eCO}_2$ levels. This information enables firefighters to assess potential risks and adapt their routes based on evolving conditions within the building. The advantage of our system is that it offers timely and actionable information, allowing firefighters to make informed decisions dynamically as conditions change. Additionally, by providing real-time predictions and insights on evolving fire conditions, our system empowers firefighters to adjust their strategies and operational tactics on the ground. This is achieved through a user-friendly interface that presents the data in an intuitive format, enabling quicker and more informed decision-making. We understand that dynamic information is already available in normal systems, but our contribution is in providing this information in a more accessible and user-friendly manner, tailored to the needs of firefighting interventions.

## Data Availability

The datasets used and/or analysed during the current study available from the corresponding author on reasonable request.
